# Three-year outcome comparison between extended early intervention and standard psychiatric care for adults with first-episode psychosis in Hong Kong

**DOI:** 10.1017/S0033291725102389

**Published:** 2025-11-05

**Authors:** Matthew Tsz Ho Ho, Ryan Sai Ting Chu, Tsz Ting Lui, Fu Chun Lau, Edwin Ho Ming Lee, Christy Lai Ming Hui, Sherry Kit Wa Chan, Eric Chen, Wing Chung Chang

**Affiliations:** 1 Department of Psychiatry, School of Clinical Medicine, LKS Faculty of Medicine, University of Hong Kong, Hong Kong SAR, China; 2 Department of Psychiatry, United Christian Hospital, Hong Kong; 3 Centre for Youth Mental Health, The University of Melbourne, Parkville, VIC, Australia

**Keywords:** early intervention, effectiveness, first-episode psychosis, psychosocial functioning

## Abstract

**Background:**

Early intervention (EI) for first-episode psychosis (FEP) mainly focuses on adolescents and young adults. Previous evaluation demonstrated superiority of 2-year EI program (EASY) over standard care in outcome improvement in young people (15–25 years) with FEP in Hong-Kong. However, effectiveness of territory-wide extended EASY, which provides 3-year EI service also to adult patients aged ≥26 years, has not been systematically examined.

**Methods:**

This study adopted historical control–case design, comparing patients aged 26–55 years who had received extended EI (EI-group, *n* = 160) with those managed by standard psychiatric care (SC-group, *n* = 160) prior to an implementation of extended EI service on a comprehensive range of outcomes encompassing duration of untreated psychosis (DUP), pathway to care, symptom severity, psychosocial functioning, subjective quality of life and service utilization over 3 years of psychiatric follow-up, using systematic medical-record review and follow-up interview assessment.

**Results:**

Our results showed that EI-group had significantly shorter DUP than SC-group. Additionally, EI-group displayed fewer average positive symptoms in the first and second year of follow-up, lower levels of negative and depressive symptoms, better global and social functioning, and higher quality of life on physical domain than SC-group at 3 years of follow-up. Our findings indicate that adult FEP patients receiving 3-year extended EI service had better clinical and functional outcomes than those managed by standard psychiatric care.

**Conclusions:**

Our results thus provide real-world evidence supporting the superiority and implementation of 3-year extended EASY program for adult FEP patients in shortening of treatment delay and improvement of symptom and functional outcomes.

## Introduction

Psychotic disorders, including schizophrenia, are a group of severe mental illnesses that affects 23.6 million people globally, and constitute a substantial disease burden worldwide (GBD Mental Disorders Collaborators, 2022). The disorders can lead to profound disruptions in a person’s psychosocial functioning (Jauhar, Johnstone, & McKenna, 2022), and are associated with elevated risk of premature mortality and physical comorbidity (Chan et al., 2023; Correll et al., 2022). To minimize the disease burden, numerous early intervention (EI) programs for psychosis have been established worldwide with an aim to improve treatment outcome (Fusar-Poli, McGorry, & Kane, 2017). This is based on the premise that shortening of treatment delay and provision of phase-specific treatments in the initial few years of psychosis can improve long-term prognosis (Salazar de Pablo, Aymerich et al., 2024; Williams et al., 2024). Accumulating evidence has shown that prolonged duration of untreated psychosis (DUP) is observed across geographic regions (Salazar de Pablo, Guinart et al., 2024), and that a longer DUP is associated with poorer clinical and functional outcomes (Howes et al., 2021).

Meta-analytic reviews have demonstrated that patients receiving EI service for psychosis had significantly better outcomes than those managed by standard psychiatric care with respect to symptom severity, functioning, hospitalization, and treatment discontinuation (Correll et al., 2018). Hong Kong (HK) is among the few cities in Asia implementing the EI service for psychosis. The EI program, namely Early Assessment Service for Young People with Psychosis (EASY), was launched in 2001 (Chen & Treffurth, 2024). EASY program was a publicly funded, territory-wide service which comprises a community awareness program, an open referral system, comprehensive evaluation, and a 2-year specialized intervention for individuals aged 15–25 years presenting with first-episode psychosis (FEP). As part of the community awareness program, a more accurate and less stigmatizing Chinese translation for the term ‘psychosis’ was introduced. A widespread public dissemination of information about the symptoms of psychosis and the need for early intervention was initiated. A hotline was set up for direct screening, enquiry and public referral as a means to facilitate early detection and service access to reduce treatment delay (Wong et al., 2012). The service has adopted a phase-specific case-management approach with multi-disciplinary inputs in which each patient was assigned a case manager who provided protocol-based psychosocial interventions according to patients’ stages of illness and needs (Chen & Treffurth, 2024). Of note, our previous study found that only around half of the patients treated in 2-year EI service achieved adequate social and occupational functioning at the end of 3 years of follow-up (Chang et al., 2018). This is consistent with the literature demonstrating that a significant proportion of patients still exhibited persistent functional impairment even in the presence of clinical remission and upon discharge from the specialized care (Lally et al., 2017; Verma et al., 2012). In addition, accumulating evidence has suggested the unmet treatment need and service gap of individuals presenting with FEP at later adulthood as EI services have mainly focused on adolescents and young adults (Clay et al., 2018; Ferrara et al., 2024; Greenfield et al., 2018; Jagger et al., 2020; Thakrar, Bardhan, & Chakraborty, 2023). In an attempt to provide a more comprehensive EI service in HK, the EASY program has been extended to provide 3-year specialized care for FEP covering a wider age range (15 to 64 years) since 2011. Our prior investigation has demonstrated superior effectiveness of 2-year EASY program over standard care on clinical and functional outcomes in young people (aged 15–25 years) presenting with FEP (Chen et al., 2011). However, whether and to what extent the extended EI service was effective in outcome improvement relative to standard care on older adult FEP patients remained to be clarified.

To this end, we conducted a historical control–case study to compare adult FEP patients who received extended EI service with those managed by standard psychiatric care on 3-year clinical and functional outcomes. Specifically, a comprehensive range of outcomes was examined, encompassing treatment delay (DUP), pathways to care, symptom severity, psychosocial functioning, subjective quality of life, and service utilization.

## Methods

### Study design

The fact that the extended EASY program is a territory-wide clinical service covering the whole of HK and the ethical concerns on withholding specialized EI for FEP patients after program expansion precluded adoption of a randomized-controlled trial (RCT) or concurrent case–control design for the current service evaluation. In this regard, we adopted a historical control–case study design to estimate the real-world impact of 3-year extended EI service (EI group) versus 3-year standard psychiatric care (SC group) in adult patients aged 26–55 years on clinical and functional outcomes. This methodological design has been applied in previous studies evaluating the effectiveness of EI service for psychosis (Chen et al., 2011; Cullberg et al., 2002; Goldberg et al., 2006; McGorry et al., 1996). In the current study, we employed both systematic medical record review and follow-up interview assessment to compare between EI and SC groups in treatment delay as well as 3-year clinical and functional outcomes.

Briefly, the extended EASY program is a publicly funded, territory-wide service providing early assessment and intervention for individuals aged 15–64 years presenting with FEP in HK. The service consists of seven clinical teams, each covering a geographically-defined catchment area and comprising a clinical team of psychiatrists, case managers, and social workers. The service adopts a phase-specific case management approach in which each FEP patient is assigned a case manager who provides protocol-based psychosocial interventions (So, 2013). FEP patients would be assertively followed up for 3 years, after which they are transferred to a generic psychiatric clinic for continuous care (Chen & Treffurth, 2024).

### Participants and setting

We identified 160 FEP patients (EI group) who were enrolled in the extended EASY program between 1 January 2012 and 30 July 2012, as well as 160 FEP patients (SC group) who entered standard psychiatric service between 1 September 2010 and 31 March 2011, from the Psychiatric Case Register of the Hospital Authority (*note*: the extended EASY program has been implemented since 1 April 2011). The HA provides publicly funded territory-wide psychiatric services covering a total of seven catchment areas in HK. Random sampling was applied to select participants for the EI and SC groups from their respective eligible patient cohorts within the specified timeframes. Diagnosis and first-episode status of each identified participant were verified by research psychiatrists using systematic medical record review. Patients were eligible for study inclusion if they were Chinese adults aged 26–55 years upon service entry who presented with a first-episode ICD-10 diagnosis of schizophrenia, schizoaffective disorder, acute and transient psychotic disorders, delusional disorder or other non-organic psychotic disorders. Exclusion criteria included substance-induced psychosis, psychotic disorders due to general medical conditions, bipolar disorder with psychotic features, depressive disorder with psychotic features, or intellectual disability. The study was approved by local institutional review boards of the University of Hong Kong and the Hospital Authority (UW 14–306), and all of the participants provided written informed consent (including an opt-out procedure to decline medical record review) before study participation.

### Systematic medical record review

Participants’ baseline and 3-year follow-up variables were obtained via systematic record review. For each participant, outpatient and inpatient medical records, and computerized clinical information from the HA medical record database were retrieved. Following the method of systematic medical record review adopted by our previous publications (Chang et al., 2012, 2018; Chen et al., 2011; Hui et al., 2013), trained research assistants acquired the data from medical files according to a protocol designed specifically for data collection in the current study. Standardized data entry forms were used to systematically extract information on service intake, treatment, and follow-up variables from medical files. Regular biweekly consensus meetings were held throughout the period of data collection to ensure strict adherence to the protocol and to resolve ambiguity in clinical information during the data acquisition process. Baseline data included socio-demographics, occupational status, and age at service entry. Treatment delay in the form of DUP was estimated at baseline. Final diagnosis of those patients who did not participate in the interview assessment at 3-year follow-up was determined by a senior research psychiatrist using systematic medical record review over a 3-year study period based on DSM-IV criteria. Symptom severity and functional levels were rated by the standardized instruments based on medical record review. Specifically, symptom measures, including positive and negative symptom levels were assessed individually from the Clinical Global Impression-Severity of Illness Scale (CGI-S) (Guy, 1976). Depressive symptoms were evaluated using Clinical Global Impression-Severity of Illness Scale: Bipolar Illness (CGI-BP) (Spearing et al., 1997). Functional outcome was evaluated by Social Occupational Functioning Assessment Scale (SOFAS) (Goldman, Skodol, & Lave, 1992). Relapse was defined as an increase in the level of positive symptoms leading to a change in antipsychotic medication management and/or psychiatric hospitalization following initial stabilization of participants’ symptoms. Engagement of full-time employment during follow-up was measured. Data on mortality and suicide were obtained. Data on service utilization over a 3-year follow-up, including psychiatric admissions, defaults of outpatient appointments, and service disengagement, were collected. Service disengagement was defined as continuous default of EASY outpatient appointments for at least three times up till the end of the 3-year service despite therapeutic need and active tracing from staff for psychiatric follow-up. Treatment characteristics, including antipsychotic dose (chlorpromazine equivalents were computed for analysis) (Gardner et al., 2010) were recorded. Symptom and functional outcome measures were evaluated on a monthly basis throughout the entire 3-year follow-up period. Inter-rater reliability exercise was conducted with intra-class correlation coefficients (ICCs) for CGI-S (positive and negative symptoms), CGI-BP (depressive symptoms), and SOFAS (functional outcome), yielding satisfactory level of concordance in these key variables (ranging between 0.71 and 0.90).

### Follow-up interview assessment

Participants selected for the study were contacted for a follow-up interview assessment 3 years after service entry. Final diagnosis of each participant was ascertained by a senior research psychiatrist using the Chinese-bilingual Structured Clinical Interview for DSM-IV (CB-SCID, So et al., 2003) supplemented with medical record review. Diagnosis of those participants who did not receive follow-up interview assessment was determined by systematic medical record review based on DSM-IV criteria. The Premorbid Adjustment Scale (PAS) was used to measure premorbid functioning (Cannon-Spoor, Potkin, & Wyatt, 1982). Age at onset of psychosis, first-episode status and DUP, which was defined as the time interval between the onset of positive psychotic symptoms and antipsychotic treatment initiation, were measured primarily on the basis of systematic medical record review to minimize recall bias, supplemented by the Interview for the Retrospective Assessment of the Onset of Schizophrenia (IRAOS) (Häfner et al., 1992). Help-seeking and referral patterns were also examined. Help-seeking delay was the time interval between the onset of positive psychotic symptoms and the first formal help-seeking action, while treatment delay referred to the time interval between the first formal help-seeking action and receipt of antipsychotic treatment. Positive and negative symptom severity was evaluated using the Positive and Negative Syndrome Scale (PANSS) (Kay, Fiszbein, & Opler, 1987). Depressive symptoms were measured by Calgary Depression Scale for Schizophrenia (CDSS) (Addington, Addington, & Maticka-Tyndale, 1993). Psychosocial functioning was measured by the SOFAS and Role Functioning Scale (RFS) (Goodman, Sewell, Cooley, & Leavitt, 1993). SOFAS provided a global functioning estimate of an individual patient, while RFS, which comprises four subscales, was used to assess functional levels of various domains, including independent living and self-care, work productivity, and immediate and extended social networks. Employment status was also obtained. The Chinese version of SF12, which has been validated and studied in previous local FEP research, was used to measure patients’ subjective quality of life (Lam et al., 2010; Lee et al., 2016). Trained research assistants who conducted the systematic medical record review for EI and SC groups were also responsible for administering the follow-up interview assessment for these two treatment groups. Inter-rater reliability exercise was conducted with ICCs for PANSS general psychopathology, positive and negative symptom subscales, and CDSS score being 0.87, 0.90, 0.78 and 0.92, respectively, indicating good inter-rater reliability. A satisfactory level of concordance was also observed in functional measures, with ICCs for SOFAS and RFS total score being 0.81 and 0.84, respectively.

### Statistical analysis

To test whether the two treatment groups were comparable at baseline, between-group differences in demographics, premorbid adjustment, clinical profiles, and functioning at service intake, and medication treatment were examined using chi-square test and independent *t*-test as appropriate. DUP, help-seeking, and referral patterns were compared between the two groups to examine the potential differential impact of treatment provision on treatment delay and pathway to care. Between-group comparisons on various outcome measures and treatment characteristics were conducted using the chi-square test and independent *t*-test as appropriate. Analysis of covariance (ANCOVA) (for continuous outcome variables) and binary logistic regression model (for categorical outcome variables) were then applied to those outcome variables that were found to be significantly different between the treatment groups in the preceding analyses, adjusting for the effect of DUP on outcomes. Due to the skewed distribution, DUP, help-seeking delay, and treatment delay were log-transformed for parametric analysis. The level of statistical significance for all analyses was set at *p* < 0.05.

## Results

### Baseline characteristics of the study sample

In total, 320 patients (160 in the EI group and 160 in the SC group) were included in the study. As shown in Table 1, no significant differences between the two groups were observed regarding demographics and baseline clinical, functional, and treatment characteristics based on systematic record review. Among the 320 patients, 251 participated in an interview assessment at the end of the 3-year follow-up. There was no significant difference in non-participation rate between the two groups for follow-up interview assessment (130 in the EI group and 121 in the SC group; *p* > 0.05). Among those non-interviewed participants (*n* = 69), 7 were deceased (including 2 who died of suicide), and the remaining 62 participants were traced but refused follow-up assessment. The causes of attrition are shown in Supplementary Table S1. As shown in Table 2, the two groups did not differ in socio-demographics, premorbid adjustment, illness characteristics, baseline symptom and functional levels, and antipsychotic medication use. The results thus indicated that the EI and SC groups were comparable at service intake. Further analyses comparing demographics, baseline characteristics, and DUP between interviewed and non-interviewed patients revealed no significant between-group differences (Supplementary Table S2).

**Table 1.** Demographics, and baseline clinical, functional, and treatment characteristics of patients based on retrospective medical record review

Variables of interest	Early intervention (*n* = 160)	Standard care (*n* = 160)	*t*/χ^2^	*p*
Demographics				
Male gender, *n* (%)	66 (41.3)	61 (38.1)	0.33	0.57
Age at service entry, mean (SD)	37.6 (8.5)	39.2 (8.0)	1.75	0.08
Full-time employed at entry, *n* (%)	20 (12.5)	27 (16.9)	1.22	0.27
Illness characteristics				
Age at psychosis onset, years, mean (SD)	36.6 (8.9)	37.3 (8.2)	−0.72	0.47
Psychiatric diagnosis, *n* (%)			2.19	0.14
Schizophrenia-spectrum disorders^a^	130 (81.2)	119 (74.4)		
Other non-affective psychoses^b^	30 (18.8)	41 (25.6)		
Baseline symptom and functional levels				
CGI-S positive symptom score, mean (SD)	5.1 (1.2)	5.4 (1.0)	−1.67	0.09
CGI-S negative symptom score, mean (SD)	2.6 (1.3)	2.7 (1.5)	−0.68	0.50
CGI-BP depression score, mean (SD)	2.0 (1.3)	1.9 (1.2)	0.83	0.41
SOFAS score, mean (SD)	41.8 (12.6)	40.7 (11.5)	0.79	0.43
Treatment characteristics				
On antipsychotic medication, *n* (%)	159 (99.4)	156 (98.1)	1.03^c^	0.37
CPZ equivalent dose, mg, mean (SD)	146.1 (102.1)	172.2 (225.2)	−1.32	0.19

Abbreviations: CGI-BP, Clinical Global Impression - Severity of Illness Scale: Bipolar illness; CGI-S, Clinical Global Impression – Severity Scale; CPZ, chlorpromazine; SOFAS, Social and Occupational Functioning Assessment Scale.

a Schizophrenia-spectrum disorders include schizophrenia, schizophreniform disorder and schizoaffective disorder.

b Other affective psychoses include brief psychotic disorder, delusional disorder and psychotic disorders not otherwise specified (NOS).

c Fisher’s exact test was applied.

**Table 2.** Demographics, premorbid profiles, and baseline clinical, functional, and treatment characteristics of patients who participated in 3-year follow-up interview assessment^a^

Variables of interest	Early intervention (*n* = 130)	Standard care (*n* = 121)	*t*/*χ* ^2^	*p*
Socio-demographics				
Male gender, *n* (%)	53 (40.8)	42 (34.7)	0.98	0.32
Age at service entry, mean (SD)	37.1 (8.4)	39.1 (8.1)	−1.90	0.07
Educational level, *n* (%)				
Secondary level	97 (74.6)	99 (81.8)	2.39^c^	0.30
Tertiary level	28 (21.5)	17 (14.1)		
Above tertiary level	5 (3.9)	5 (4.1)		
Full-time employed at entry, *n* (%)	41 (31.5)	42 (34.7)	0.29	0.59
Premorbid function and family history				
PAS overall score, mean (SD)	0.3 (0.2)	0.4 (0.2)	−1.85	0.07
Family history of psychotic disorder, *n* (%)	31 (24.0)	26 (21.9)	0.17	0.68
History of substance abuse, *n* (%)	5 (3.9)	6 (5.0)	1.28	0.53
Illness characteristics				
Age at psychosis onset, years, mean (SD)^b^	36.0 (8.7)	37.2 (8.5)	−1.08	0.28
Psychiatric diagnosis, *n* (%)^c^			0.84	0.36
Schizophrenia-spectrum disorders^d^	106 (81.5)	93 (76.9)		
Other non-affective psychoses^e^	24 (18.5)	28 (23.1)		
Baseline symptom and functional levels				
CGI-S positive symptom score, mean (SD)	5.2 (1.1)	5.2 (1.1)	−0.03	0.98
CGI-S negative symptom score, mean (SD)	2.7 (1.3)	2.7 (1.5)	−0.04	0.97
CGI-BP depression score, mean (SD)	2.0 (1.3)	2.0 (1.2)	−0.35	0.73
SOFAS score, mean (SD)	40.8 (12.1)	41.4 (11.7)	−0.38	0.70
Treatment characteristics				
On antipsychotic medication, *n* (%)	129 (99.2)	119 (99.2)	0.00^f^	1.00
CPZ equivalent dose, mg, mean (SD)	145.8 (101.3)	179.4 (242.4)	−1.40	0.16

Abbreviations: CGI-BP, Clinical Global Impression - Severity of Illness Scale: Bipolar illness; CGI-S, Clinical Global Impression – Severity Scale; CPZ, chlorpromazine; PAS, Premorbid Adjustment Scale; SOFAS, Social and Occupational Functioning Assessment Scale.

a Variables were obtained from the interview assessment at 3-year follow-up, except those of the baseline symptom and functional levels and treatment characteristics.

b Age at psychosis onset was primarily based on medical records at baseline, supplemented by the interview assessment at 3-year follow-up.

c Final diagnosis of each participant was ascertained using the Chinese-bilingual Structured Clinical Interview for DSM-IV supplemented with medical record review.

d Schizophrenia-spectrum disorders included schizophrenia, schizophreniform disorder and schizoaffective disorder.

e Other affective psychoses included brief psychotic disorder, delusional disorder and psychotic disorders not otherwise specified (NOS).

f Fisher’s exact test was applied.

### Outcome comparisons between EI and SC groups based on medical record review

Table 3 summarizes the results of comparison analyses between EI and SC groups on treatment delay and 3-year clinical and functional outcomes. Patients in the EI group had significantly shorter DUP than those in the SC group, though no significant between-group difference was observed with respect to the proportion of patients receiving inpatient treatment upon service entry. Regarding symptom outcomes, patients in the EI group had significantly lower average CGI-S positive symptom levels in the first and second year of follow-up than patients in SC group. Patients in EI group also attained significantly higher average SOFAS scores in the first, second and third year of follow-up, and were engaged in full-time work for a longer period of time over the 3-year treatment period than those in the SC group. There was a trend-wise significance showing that EI patients were less likely than SC patients to disengage from psychiatric service over 3-year follow-up (and had longer length of service stay). The two groups demonstrated no significant differences in rates of relapse, all-cause mortality, and completed suicides. No significant between-group differences were observed in hospitalization outcomes and outpatient default rate. After controlling for the effect of DUP, the superiority of EI group over SC group with respect to 1-year (*F* = 12.6, *p* < 0.001) and 2-year CGI-S positive symptom severity (*F* = 4.3, *p* = 0.04), and 1-year (*F* = 5.8, *p* = 0.02), 2-year (*F* = 11.6, *p* = 0.001) and 3-year SOFAS scores (*F* = 17.8, *p* = <0.001) still remained statistically significant (Supplementary Table S3).

**Table 3.** Treatment delay and 3-year outcome comparisons between the two treatment groups based on retrospective medical record review

Variables of interest	Early intervention	Standard care	*t*/χ^2^	*P*
Service entry status and treatment delay				
Inpatient treatment upon service entry, *n* (%)	78 (48.8)	91 (56.9)	2.12	0.15
DUP, days, mean (SD)	353.7 (545.9)	620.7 (1045.8)	–	–
Log DUP^a^ ^,^^b^, mean (SD)	2.0 (0.8)	2.3 (0.8)	−2.40	0.017*
Symptom outcome^c^				
First year^d^ average score, mean (SD)				
CGI-S positive symptom score	2.2 (0.9)	2.7 (1.2)	−4.04	<0.001***
CGI-S negative symptom score	2.1 (1.1)	2.3 (1.2)	−1.42	0.16
CGI-BP depression score	1.4 (0.7)	1.5 (0.7)	−0.85	0.40
Second year^e^ average score, mean (SD)				
CGI-S positive symptom score	1.4 (0.7)	1.7 (0.9)	−2.46	0.02*
CGI-S negative symptom score	1.8 (1.1)	2.0 (1.1)	−1.65	0.10
CGI-BP depression score	1.2 (0.5)	1.2 (0.5)	−1.48	0.14
Third year^f^ average score, mean (SD)				
CGI-S positive symptom score	1.5 (0.8)	1.6 (0.9)	−1.07	0.29
CGI-S negative symptom score	1.7 (1.1)	1.9 (1.1)	−1.64	0.10
CGI-BP depression score	1.1 (0.5)	1.2 (0.5)	−1.20	0.23
Functional outcome^c^				
First year^d^ average SOFAS, mean (SD)	51.2 (9.6)	48.2 (9.6)	2.66	0.01**
Second year^e^ average SOFAS, mean (SD)	56.2 (9.9)	52.0 (9.6)	3.72	<0.001***
Third year^f^ average SOFAS, mean (SD)	57.4 (10.3)	52.1 (9.3)	4.53	<0.001***
Months in full-time work in 3 years^f^, mean (SD)	11.5 (14.0)	8.0 (12.7)	2.34	0.02*
Other clinical outcome variables over 3 years				
Relapse of psychotic episodes, *n* (%)	61 (38.1)	51 (31.9)	0.55	0.46
All-cause mortality, *n* (%)	2 (1.3)	5 (3.1)	1.31^g^	0.45
Suicide, *n* (%)	2 (1.3)	2 (1.3)	0.00^g^	1.00
Service utilization over 3 years				
Psychiatric hospitalization, *n* (%)	44 (27.5)	46 (28.7)	0.19	0.67
Length of hospital stay, days, mean (SD)	13.5 (40.1)	16.4 (45.3)	−0.57	0.57
Default in outpatient appointment, *n* (%)	67 (41.9)	72 (45.0)	0.32	0.57
Service disengagement, *n* (%)^h^	17 (10.6)	28 (17.5)	3.13	0.08
Length of service stay, months, mean (SD)	33.8 (7.6)	31.8 (10.1)	1.92	0.06
Treatment characteristics				
CPZ equivalent dose at 1 year, mg, mean (SD)	306.5 (218.7)	299.6 (221.1)	0.26	0.80
CPZ equivalent dose at 2 years, mg, mean (SD)	338.5 (267.3)	312.6 (216.2)	0.85	0.40
CPZ equivalent dose at 3 years, mg, mean (SD)	351.0 (327.5)	353.0 (293.7)	−0.05	0.96

Abbreviations: CGI-BP, Clinical Global Impression - Severity of Illness Scale: Bipolar illness; CGI-S, Clinical Global Impression – Severity Scale; CPZ, chlorpromazine; DUP, Duration of untreated psychosis; SOFAS, Social and Occupational Functioning Assessment Scale.

a DUP was log-transformed for parametric analysis owing to its skewed distribution.

b The median DUP of early intervention and standard care groups were 118 and 306 days, respectively.

c Monthly symptom and functioning rating method and calculation of the yearly average scores: If a patient had more than one outpatient follow-up visit within a month (e.g. 2 visits a month), we rated CGI-S/CGI-BP/SOFAS with the most severe symptom/lowest functional levels for the ratings of that month. If a patient had less frequent outpatient follow-up visit, e.g. 1 visit in 3 months, then the patient would be assigned with the same symptom/functioning ratings for the three consecutive months until the patient had next outpatient visit. We calculated the mean CGI-S/CGI-BP/SOFAS ratings by dividing the sum of the symptom/functioning ratings by 12 months per year to derive yearly average symptom/functioning score.

d 151 patients of early intervention group and 141 patients of standard care group completed 1-year service and were included in analysis.

e 148 patients of early intervention group and 134 patients of standard care group completed 2-year service and were included in analysis.

f 143 patients of early intervention group and 132 patients of standard care group completed 3-year service and were included in analysis.

g Fisher’s exact test was applied. *P < 0.05, **P < 0.01, ***P < 0.001

h Service disengagement was defined as continuous default of psychiatric outpatient appointments for at least 3 times up till the end of the 3-year service despite therapeutic need and active tracing from staff for psychiatric follow-up.

### Outcome comparisons between EI and SC groups based on follow-up interview assessment

As shown in Table 4, patients in the EI group did not differ from those in the SC group regarding patterns of first help-seeking action and referral source. EI group displayed shorter DUP, help-seeking delay, and referral delay than the SC group, but did not reach statistical significance. Upon follow-up assessment, EI patients demonstrated significantly less severe positive symptoms, negative symptoms, general psychopathology, and depressive symptoms than SC patients. EI patients also displayed significantly higher RFS total score, RFS immediate and extended social network scores, as well as SF12 physical domain score than SC patients. There were no significant group differences in treatment characteristics, including antipsychotic-induced motor side-effects and medication dose. As patients in the EI group had shorter DUP (albeit statistically non-significant in this interviewed FEP sample) than those in the SC group, alongside the fact that EI patients had significantly shorter DUP than SC patients based on data from record review, a series of ANCOVAs were applied, with DUP as a covariate, for further outcome analysis. EI patients still had better outcomes than SC patients in PANSS negative symptoms (*F* = 7.8, *p* = 0.01) and general psychopathology (*F* = 30.1, *p* < 0.001), CDSS score (*F* = 4.9, *p* = 0.03), total RFS score (*F* = 4.2, *p* = 0.04), RFS immediate (*F* = 5.9, *p* = 0.02) and extended social network scores (*F* = 6.0, *p* = 0.02), and SF12 physical domain score (*F* = 5.3, *p* = 0.02), with the exception of positive symptom severity (*F* = 3.3, *p* = 0.07) (Supplementary Table S4).

**Table 4.** Treatment delay and outcome comparisons between the two treatment groups based on interview assessment

Variables of interest	Early intervention (*n* = 130)	Standard care (*n* = 121)	*t*/*χ* ^2^	*P*
Pathway to care and treatment delay				
First help-seeking action, *n* (%)			0.97	0.62
Community non-medical sector^a^	49 (38.0)	39 (32.5)		
Community medical sector^b^	34 (26.4)	32 (26.7)		
Non-community medical sector^c^	46 (35.6)	49 (40.8)		
Referral source, *n* (%)			4.66	0.10
Community non-medical sector	8 (6.3)	17 (14.1)		
Community medical sector	20 (15.9)	22 (18.2)		
Non-community medical sector	98 (77.8)	82 (67.7)		
Inpatient status upon service entry, *n* (%)	70 (53.9)	69 (48.8)	0.26	0.61
DUP, days, mean (SD)	375.8 (565.8)	635.2 (1136.1)	–	–
Log DUP^d^ ^,^^e^, mean (SD)	2.1 (0.8)	2.2 (0.8)	−1.54	0.13
Help-seeking delay, days, mean (SD)	330.7 (534.4)	67.4 (1088.3)	–	–
Log help-seeking delay^e^, mean (SD)	2.0 (0.8)	2.1 (0.9)	−1.29	0.20
Treatment delay, days, mean (SD)	45.7 (118.4)	68.5 (286.4)	–	–
Log treatment delay^e^, mean (SD)	0.7 (0.9)	0.7 (0.9)	−0.36	0.72
Symptom severity at follow-up				
PANSS positive symptom score, mean (SD)	8.7 (3.2)	9.6 (3.8)	−2.13	0.03*
PANSS negative symptom score, mean (SD)	10.8 (5.7)	13.4 (8.0)	−2.91	0.01*
PANSS general psychopathology, mean (SD)	19.4 (4.0)	24.4 (8.9)	−5.59	<0.001***
CDSS score, mean (SD)	1.8 (3.0)	2.8 (3.7)	−2.28	0.02*
Functional outcome at follow-up				
SOFAS score, mean (SD)	59.0 (11.4)	57.3 (12.1)	1.16	0.25
RFS total score, mean (SD)	22.4 (3.3)	21.3 (4.0)	2.30	0.02*
RFS work productivity, mean (SD)	5.1 (1.9)	4.9 (1.9)	0.65	0.52
RFS independent living, mean (SD)	6.8 (0.5)	6.6 (1.0)	1.94	0.06
RFS immediate social network, mean (SD)	5.7 (1.1)	5.4 (1.2)	2.70	0.01*
RFS extended social network, mean (SD)	4.7 (0.9)	4.4 (1.2)	2.59	0.01*
Full-time employed at follow-up, *n* (%)	41 (31.5)	42 (34.7)	0.29	0.59
Subjective quality of life at follow-up				
SF12 physical domain score, mean (SD)	49.0 (7.1)	46.4 (8.1)	2.47	0.01*
SF12 mental domain score, mean (SD)	48.0 (9.2)	46.5 (11.7)	1.06	0.29
Treatment characteristics and side-effects				
On antipsychotic medication, *n* (%)	122 (93.8)	112 (93.3)	0.03	0.87
CPZ equivalents at follow-up, mg, mean (SD)	416.0 (595.3)	387.2 (307.4)	0.46	0.65
SAS average score	0.2 (1.0)	0.5 (1.6)	−1.64	0.10
BARS global score	0.1 (0.5)	0.2 (0.7)	−1.29	0.20
AIMS total score	0.3 (1.8)	0.3 (1.1)	0.27	0.79

Note: *P < 0.05, **P < 0.01, *** P < 0.001.

Abbreviations: AIMS, Abnormal Involuntary Movement Scale; BARS, Barnes Akathisia Rating Scale; CDSS, Calgary Depression Scale for Schizophrenia; CPZ, chlorpromazine; DUP, Duration of untreated psychosis; PANSS, Positive and Negative Syndrome Scale; RFS, Role Functioning Scale; SAS, Simpson-Angus Scale; SF12, 12-Item Short Form Health Survey; SOFAS, Social and Occupational Functioning Assessment Scale.

a Community non-medical sector included social workers, schools, clinical psychologist/counsellor, traditional healers, religious priests and police. Family members/friends or self were also included in the Referral sources.

b Community medical sector included general practitioners (private or public sectors), private psychiatrists, and specialists of other medical specialties.

c Non-community medical sector included Emergency department, consultation liaison and other medical specialities. Outpatient psychiatric unit other than EASY program was also included in the Referral source.

d The median DUP for early intervention and standard care groups were 144 and 273 days, respectively.

e Log-transformation was employed for parametric analysis owing to skewed distribution of the data.

## Discussion

The current study examined the effectiveness of a territory-wide specialized extended EI program on the reduction of treatment delay and improvement of clinical and functional outcomes in adult Chinese FEP patients in HK by adopting a historical control–case design to compare patients who received 3-year EI service with those managed by standard care, with outcome evaluation comprising both systematic record review and follow-up interview assessment. Our results indicated that the EI group had significantly shorter DUP, low levels of symptom severity, and better functional outcome than the SC group over a 3-year follow-up.

One major target of the EI service is to reduce treatment delay. In fact, substantial evidence has consistently demonstrated that prolonged DUP is associated with worse short-term as well as longer-term clinical and functional outcomes in FEP patients (Howes et al., 2021). Our results that patients presenting with FEP to extended EI service had shorter DUP than those entering standard psychiatric care concur with some, though not all previous studies evaluating the impact of EI service on shortening of treatment delay (Murden, Allan, Hodgekins, & Oduola, 2024). We also found that EI patients demonstrated shorter help-seeking and referral delays than SC patients, albeit not reaching statistical significance. Nonetheless, patterns of both the first formal help-seeking action and referral source were similar between the EI and SC groups. It is plausible that the EI service for younger individuals that has been implemented prior to the extension of EASY program has contributed to raised awareness of psychosis within the community. Such increased public awareness might positively influence the help-seeking behaviors and shorten referral delays in SC patients, thereby resulting in a non-significant difference in both help-seeking and referral delays between the two groups. Taken together, our findings indicated that the extended EASY program was effective in shortening overall treatment delay but might be inadequate in lowering the risk of exposure to the negative pathway to care at first presentation (Gronholm, Thornicroft, Laurens, & Evans-Lacko, 2017).

Our findings that EI patients displayed significantly better functional outcome during the 3-year treatment period and at follow-up assessment than SC patients accord with the literature, which has shown that EI is superior to standard care in improving functional outcome (Correll et al., 2018). Importantly, the benefits of EI care on better functional outcome remained significant even after the effect of DUP was adjusted for, indicating that the superiority of EI over SC is independent of the shortening of treatment delay. In particular, we replicate the positive findings of our previous EASY evaluation studies in young FEP patients (Chang et al., 2017; Chen et al., 2011) and extend the empirical evidence of the superiority of EI over SC on functional enhancement to adult FEP participants (Hui et al., 2023). Notably, similar to many EI services, the incorporation of case management and psychosocial interventions, such as caregiver psychoeducation, social support, and work rehabilitation, in the EASY program is identified as a core component of EI services for people with psychosis to achieve sustained clinical benefits (Williams et al., 2024). It should, however, be noted that patients in both treatment groups still displayed a moderate degree of functional disability after completing 3-year treatment (based on functional ratings of both record review and interview assessment). Although patients in the EI group exhibited better functional outcome, they only attained an average SOFAS score of <60 at the end of 3-year follow-up, indicating an inadequate level of psychosocial functioning. Our results thus concur with accumulating evidence that showed that early psychosis patients still experienced significant functional impairment even when clinical stabilization was achieved (Lally et al., 2017). Our findings that EI patients attained higher ratings in SF12 (especially in the physical domain) are consistent with several prior investigations, which showed that FEP patients receiving EI care had better subjective quality of life than those in standard care (Garety et al., 2006; Kane et al., 2016).

Consistent with many previous studies examining the effect of specialized EI service on symptom outcomes in FEP populations (Chang et al., 2017; Chen et al., 2011; Nordentoft et al., 2014), our findings demonstrated that EI patients displayed significantly lower levels of negative and depressive symptoms after 3-year extended EI care than SC patients, even when the effect of reduced DUP was taken into consideration. This is of critical clinical significance, as negative symptoms are associated with poor functional outcome (Galderisi, Mucci, Buchanan, & Arango, 2018; Marder & Umbricht, 2023) and limited response to pharmacotherapy (Galderisi et al., 2021). Research has also demonstrated that depressive symptoms frequently occur in people with psychotic disorders (Chang, Cheung et al., 2015; Edwards, Garety, & Hardy, 2019) and are closely associated with heightened suicide risk, particularly in the early stage of the illness (Chang, Chen et al., 2015; Coentre, Talina, Góis, & Figueira, 2017). Alternatively, our results of better outcomes on positive symptoms in the first 2 years of extended EI treatment based on medical record review and a trend-wise significance (after DUP effect was adjusted) of lower PANSS positive symptom scores in EI patients at follow-up interview assessment are in line with our previous evaluation of EASY program for young FEP patients which revealed fewer positive symptoms in EI patients (Chen et al., 2011).

Contrary to a number of past EI evaluation studies demonstrating that patients who received EI service had fewer admissions than those in standard care (Srihari et al., 2015; Valencia, Juarez, & Ortega, 2012), we did not find significant differences between EI and SC patients in hospitalization rate and length of inpatient stay over 3-year treatment period. It is plausible that an absence of group difference in relapse rate during follow-up may partly explain the null finding on psychiatric hospitalizations. The discrepant result may also be attributable to the difference in caseloads between our extended EASY program and EI services of some Western countries, with the former having a significantly higher patient-to-case manager ratio that may otherwise lower the capacity of our EI service in reducing the risk of rehospitalization. Thus, our low-resource, high-caseload EI service would likely compromise the treatment intensity of the case management, which may lead to less effective relapse prevention and crisis intervention for patients with acute exacerbation of psychotic symptoms in the community, resulting in increased rehospitalization rates. Alternatively, the modest sample size of our study may limit its statistical power to properly examine the treatment effects of extended EI service on readmission. Likewise, this methodological constraint is equally applied to an evaluation of mortality and suicide rates that require an even larger sample size due to its rarity of occurrence. In agreement with most prior EI research (Kane et al., 2016; Nordentoft et al., 2014), we found that EI patients had a lower rate of service disengagement than SC patients.

Several methodological limitations warrant consideration in interpreting the study results. First, owing to the constraint of study design, interview assessment could not be conducted at service intake. Thus, patients’ data regarding clinical, functional, and service utilization variables at baseline and across 3-year treatment period relied on retrospective medical record review. Although several quality-control measures were implemented, data retrieved from and assessed based on record review may be biased by varying degrees of documentation quality. Symptom and functioning assessments conducted by record review were also less refined and comprehensive than interview-based evaluations. Furthermore, other important outcome variables, such as subjective quality of life, could not be addressed by record review. Second, measurement of treatment delay is inherently retrospective with potential recall bias. This was partly addressed by employing a structured interview incorporating multiple sources of information from patients and caregivers, and systematic medical record review to improve the reliability of DUP evaluation. Third, outcomes on hospitalization should be treated with caution, as the current study was likely underpowered in examining these variables. Fourth, it is worth noting that there is substantial variation in EI programs across different regions regarding the content and treatment intensity of service provided, as well as socio-cultural and mental healthcare contextual factors. Given that the findings of the current study were based on EI service of comparatively low resources and high caseloads relative to those well-established early psychosis programs implemented in some Western countries, our results should be generalized to other populations with caution.

In conclusion, our study demonstrated superior efficacy of the extended EI service over standard psychiatric care in reducing treatment delay, improving negative and depressive symptom outcomes, and enhancing functional levels in Chinese adult FEP patients. To further reduce the rates of psychiatric admission and enhance illness outcome, the treatment intensity and components of the extended EI program should be optimized, such as lowering the patient-to-case-manager ratio. In addition, future evaluation should examine the sustainability of positive effects, cost-effectiveness of the interventions, therapeutic effects in different age groups, and differential effects of individual intervention components of the EI service on illness outcomes, in order to inform further development and enhancement of the EI service model for psychosis.

## Supporting information

Ho et al. supplementary material Ho et al. supplementary material

## Data Availability

The data that support the findings of this study are available from the corresponding author upon reasonable request.

## Supplementary material

The supplementary material for this article can be found at http://doi.org/10.1017/S0033291725102389.
