# Supplementary material for: Three-year outcome comparison between extended early intervention and standard psychiatric care for adults with first-episode psychosis in Hong Kong
Source: Psychol Med. 2025 Nov 5;55:e335. doi: 10.1017/S0033291725102389 (PMC13058664; doi:10.1017/S0033291725102389)
Supplement: Ho et al. supplementary material [file S0033291725102389sup001.docx]

**Supplementary materials**

**Table S1.** Causes of attrition in early intervention and standard care groups.

**Table S2.** Attrition analysis comparing demographic and baseline characteristics between interviewed and non-interviewed patients at follow-up.

**Table S3.** Analyses of covariance (ANCOVA) adjusting for the effect of Log DUP in outcomes based on medical record review.

**Table S4.** Analyses of covariance (ANCOVA) adjusting for the effect of Log DUP in outcomes based on interview assessment.

**Table S1.** Causes of attrition in early intervention and standard care groups.

| Causes of attrition | Early intervention  (n=30) | Standard care  (n=39) |
| --- | --- | --- |
| Disengaged despite clinical need | 14 | 23 |
| Agreed termination | 4 | 2 |
| Defaulted | 7 | 0 |
| Follow-up at private clinic | 2 | 1 |
| Deceased | 2 | 5 |
| Migration | 1 | 0 |
| Refusal | 0 | 8 |
| Total | 30 | 39 |

**Table S2.** Attrition analysis comparing demographic and baseline characteristics between interviewed and non-interviewed patients at follow-up.

| Variables of interest | Interviewed patients  (n=251) | Non-interviewed patients  (n=69) | t/χ^2^ | *P* |
| --- | --- | --- | --- | --- |
| Demographics |  |  |  |  |
| Male gender, n (%) | 99 (39.4) | 28 (40.6) | 0.03 | 0.86 |
| Age at service entry, mean (SD) | 38.0 (8.3) | 39.8 (8.2) | -1.58 | 0.12 |
| Full-time employed at entry, n (%) | 35 (13.9) | 12 (17.4) | 0.51 | 0.47 |
| Clinical characteristics |  |  |  |  |
| Psychiatric diagnosis, n (%) |  |  | 1.46^e^ | 0.23 |
| Schizophrenia-spectrum disorders^a^ | 199 (79.3) | 50 (72.5) |  |  |
| Other non-affective psychoses^b^ | 52 (20.7) | 19 (27.5) |  |  |
| Log DUP^c,d^, mean (SD) | 2.2 (0.8) | 2.1 (0.8) | 0.24 | 0.81 |
| Baseline symptom & functional levels |  |  |  |  |
| CGI-S positive symptom, mean (SD) | 5.2 (1.1) | 5.3 (1.2) | -0.30 | 0.76 |
| CGI-S negative symptom, mean (SD) | 2.7 (1.4) | 2.4 (1.3) | 1.92 | 0.06 |
| CGI-BP depression, mean (SD) | 2.0 (1.3) | 1.8 (1.2) | 1.06 | 0.29 |
| SOFAS score, mean (SD) | 41.1 (11.9) | 42.0 (12.4) | -0.60 | 0.55 |
| Treatment characteristics |  |  |  |  |
| On antipsychotic medication, n (%) | 248 (99.2) | 67 (97.1) | 1.92 | 0.21 |
| CPZ equivalent dose, mg, mean (SD) | 162.0 (183.8) | 148.5 (136.9) | 0.56 | 0.58 |

Note: CGI-BP, Clinical Global Impression - Severity of Illness Scale: Bipolar illness; CGI-S, Clinical Global Impression – Severity Scale; CPZ, chlorpromazine; SOFAS, Social and Occupational Functioning Assessment Scale

^a^ Schizophrenia-spectrum disorders include schizophrenia, schizophreniform disorder and schizoaffective disorder.

^b^ Other affective psychoses include brief psychotic disorder, delusional disorder and psychotic disorders not otherwise specified (NOS).

^c^ DUP was log-transformed for parametric analysis owing to its skewed distribution.

^d^ The median DUP of both interviewed and non-interviewed groups was 183 days.

**Table S3.** Analyses of covariance (ANCOVA) adjusting for the effect of Log DUP in outcomes based on medical record review.

| Domains | Outcome variables | Early intervention | Standard care | F | *P* |
| --- | --- | --- | --- | --- | --- |
| Symptom outcome | First-year^a^ CGI-S positive symptom score | 2.2 (0.9) | 2.7 (1.2) | 12.64 | <0.001*** |
|  | Second-year^b^ CGI-S positive symptom score | 1.4 (0.7) | 1.7 (0.9) | 4.33 | 0.04* |
| Functional outcome | First year^a^ average SOFAS, mean (SD) | 51.2 (9.6) | 48.2 (9.6) | 5.82 | 0.02* |
|  | Second year^b^ average SOFAS, mean (SD) | 56.2 (9.9) | 51.9 (9.6) | 11.58 | 0.001** |
|  | Third year^c^ average SOFAS, mean (SD) | 57.4 (10.3) | 52.1 (9.3) | 17.76 | <0.001*** |
|  | Months in full-time work in 3 years^c^, mean (SD) | 11.5 (14.0) | 8.0 (12.7) | 3.79 | 0.05 |

Note: CGI-S, Clinical Global Impression – Severity Scale; DUP, Duration of untreated psychosis; SD, standard deviation; SOFAS, Social and Occupational Functioning Assessment Scale

^a^151 patients of early intervention group and 141 patients of standard care group completed 1-year service and were included in analysis.

^b^ 148 patients of early intervention group and 134 patients of standard care group completed 2-year service and were included in analysis.

^c^ 143 patients of early intervention group and 132 patients of standard care group completed 3-year service and were included in analysis.

**P*<0.05, ***P*<0.01, ****P*<0.001

**Table S4.** Analyses of covariance (ANCOVA) adjusting for the effect of Log DUP in outcomes based on interview assessment.

| Domains | Outcome variables | Early intervention  (n=160) | Standard care  (n=160) | F | *P* |
| --- | --- | --- | --- | --- | --- |
| Symptom outcome | PANSS positive symptom score, mean (SD) | 8.7 (3.2) | 9.6 (3.8) | 3.30 | 0.07 |
|  | PANSS negative symptom score, mean (SD) | 10.8 (5.7) | 13.4 (8.0) | 7.84 | 0.01** |
|  | PANSS general symptom score, mean (SD) | 19.4 (4.0) | 24.4 (8.9) | 30.11 | <0.001*** |
|  | CDSS score, mean (SD) | 1.8 (2.9) | 2.8 (3.7) | 4.90 | 0.03* |
|  |  |  |  |  |  |
| Functional outcome | RFS total score, mean (SD) | 22.4 (3.3) | 21.3 (4.0) | 4.24 | 0.04* |
|  | RFS immediate social network, mean (SD) | 5.7 (1.1) | 5.4 (1.2) | 5.94 | 0.02* |
|  | RFS extended social network, mean (SD) | 4.7 (0.9) | 4.4 (1.2) | 6.04 | 0.02* |
|  | SF12 physical domain score, mean (SD) | 49.0 (7.1) | 46.4 (8.1) | 5.27 | 0.02* |

Note: CDSS, Calgary Depression Scale for Schizophrenia; DUP, Duration of untreated psychosis; PANSS, Positive and Negative Syndrome Scale; RFS, Role Functioning Scale; SD, standard deviation; SF12, 12-Item Short Form Health Survey

**P*<0.05, ***P*<0.01, ****P*<0.001
